# Supplementary material for: Structural remodeling of ribosome associated Hsp40-Hsp70 chaperones during co-translational folding
Source: Nat Commun. 2022 Jun 14;13:3410. doi: 10.1038/s41467-022-31127-4 (PMC9197937; doi:10.1038/s41467-022-31127-4)
Supplement: Supplementary file 1 — Supplementary Information [file 41467_2022_31127_MOESM1_ESM.pdf]

Supplementary Information

Structural remodeling of ribosome associated Hsp40-Hsp70 chaperones during co-translational folding

Yan Chen[1,2], Bin Tsai[2], Ningning Li[2], Ning Gao[2,*]

Affiliations:

[1] State Key Laboratory of Membrane Biology, School of Life Science, Tsinghua University, Beijing 100084, China.

[2] State Key Laboratory of Membrane Biology, Peking-Tsinghua Joint Center for Life Sciences, School of Life Sciences, Peking University, Beijing 100871, China.

*Correspondence to: gaon@pku.edu.cn

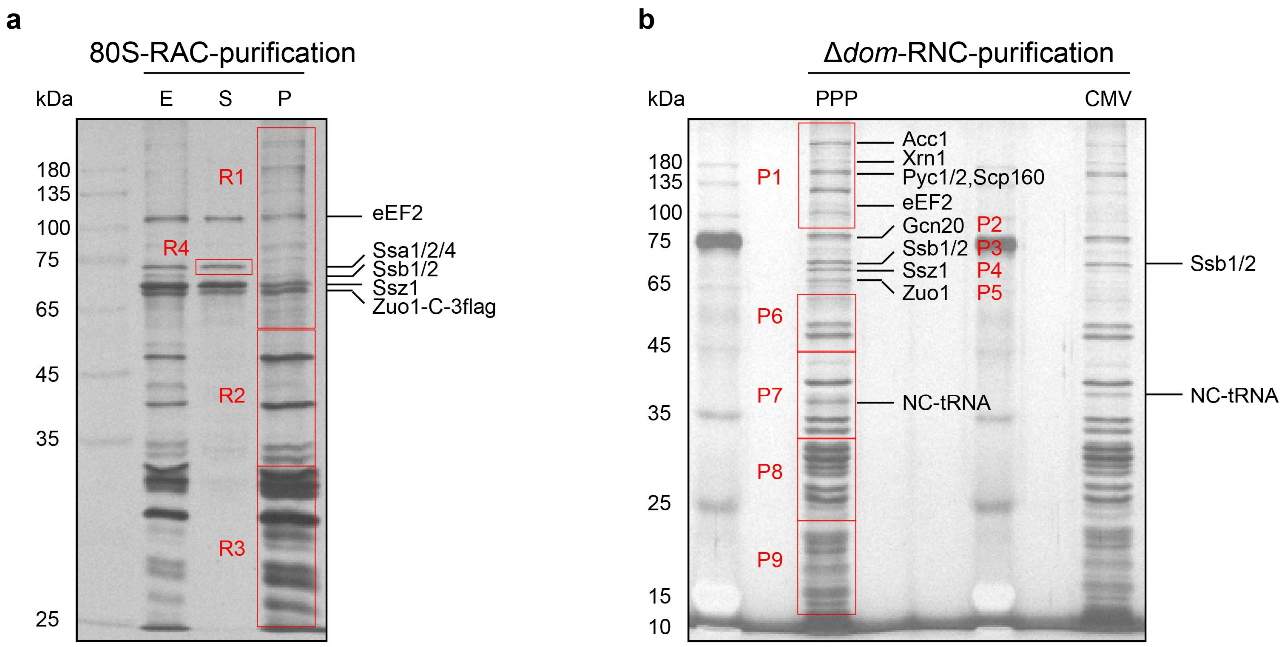

Supplementary Figure 1. Purification of the endogenous 80S-RAC and RNC-RAC complexes.

a, Silver staining of 10% PAGE gel of the endogenous 80S-RAC sample. E, elution; S and P, supernatant and pellet of the elution after a sucrose-cushion based ultracentrifugation. R1-R4 indicate the areas analyzed by mass spectrometry.

b, Silver staining of 12% PAGE gel of the RNC-RAC sample. PPP and CMV, parallel purification samples from Δ*dom*-PPP and Δ*dom*-CMV strains. NC-tRNA, nascent chain-tRNA. P1 and P6-P9 indicate the areas analyzed by mass spectrometry. P2-P5 indicate four individual bands analyzed by mass spectrometry.

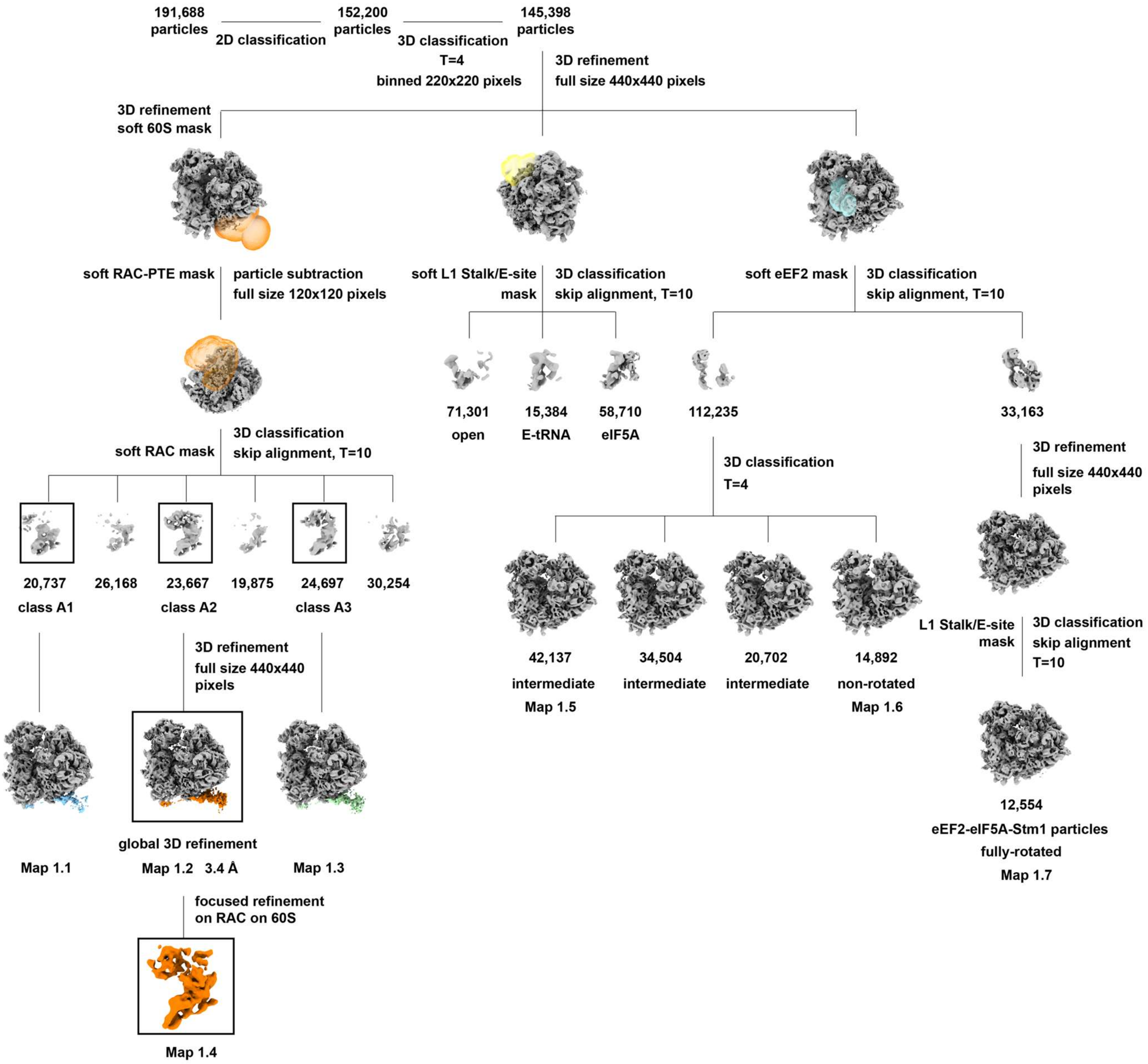

Supplementary Figure 2. Particle classification and refinement of the endogenous 80S-RAC dataset. Image processing was performed with RELION3. 2D classification was used to discard non-ribosomal or incomplete particles. See methods for details.

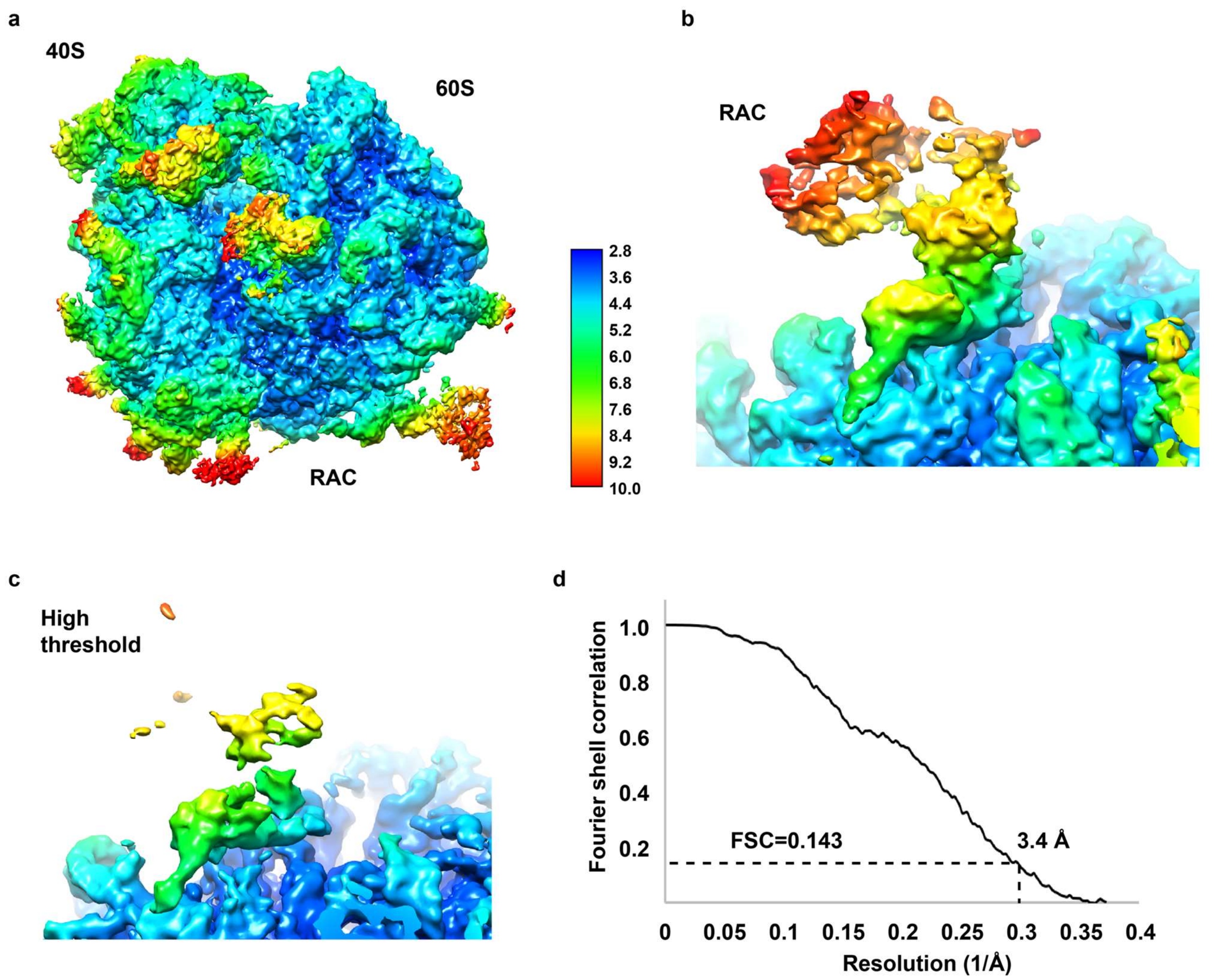

Supplementary Figure 3. Local resolution and FSC curve of the cryo-EM map of the endogenous 80S-RAC.

a, Local resolution map of State A2 of the endogenous 80S-RAC complex (Map 1.2 in Supplementary Fig. 2).

b-c, Magnified view of the density of RAC at the PTE showing at two different thresholds. The overall resolution for the ribosome is well below 4 Å, and RAC displays high flexibility indicated by a gradient of local resolution ranging from 4 to 10 Å.

d, Fourier Shell Correlation (FSC) curve for the map of State A2 (3.4 Å) using the gold standard FSC criteria cutoff (FSC=0.143).

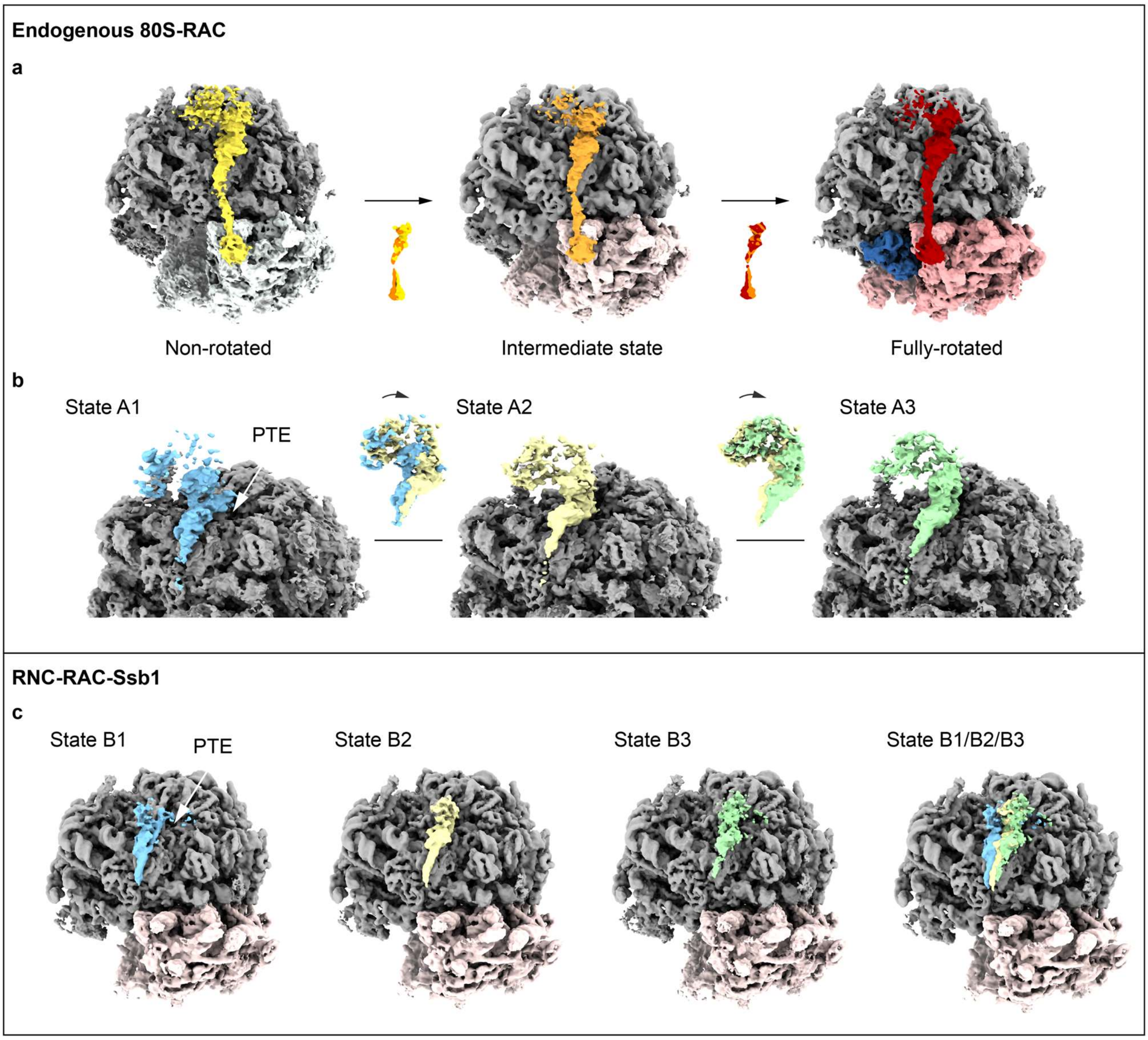

Supplementary Figure 4. Structural dynamics of RAC on the ribosomes in different conformations

a, Cryo-EM maps of the endogenous 80S-RAC complexes in differently rotated conformational states (Map 1.5, Map 1.6 and Map 1.7 in Supplementary Fig. 2). As shown, the bending of Zuo1 MD is also different. Non-rotated, intermediate state and fully-rotated 40S subunits are shown in white (left), misty rose (middle) and pink (right) and the corresponding RAC are in yellow, orange and red, respectively.

b, Dynamics of RAC at the PTE region in the endogenous 80S-RAC structures (Map 1.1, Map 1.2 and Map 1.3 in Supplementary Fig. 2). Three representative states of RAC are shown in sky blue (A1), light yellow (A2) and light green (A3). The white arrow in the left panel indicates the PTE.

c, Dynamics of RAC at the PTE region in structures from the RNC-RAC-Ssb1 dataset (Map 2.1, Map 2.2 and Map 2.3 in Supplementary Fig. 6). Three representative states of RAC are shown in sky blue (B1), light yellow (B2) and light green (B3).

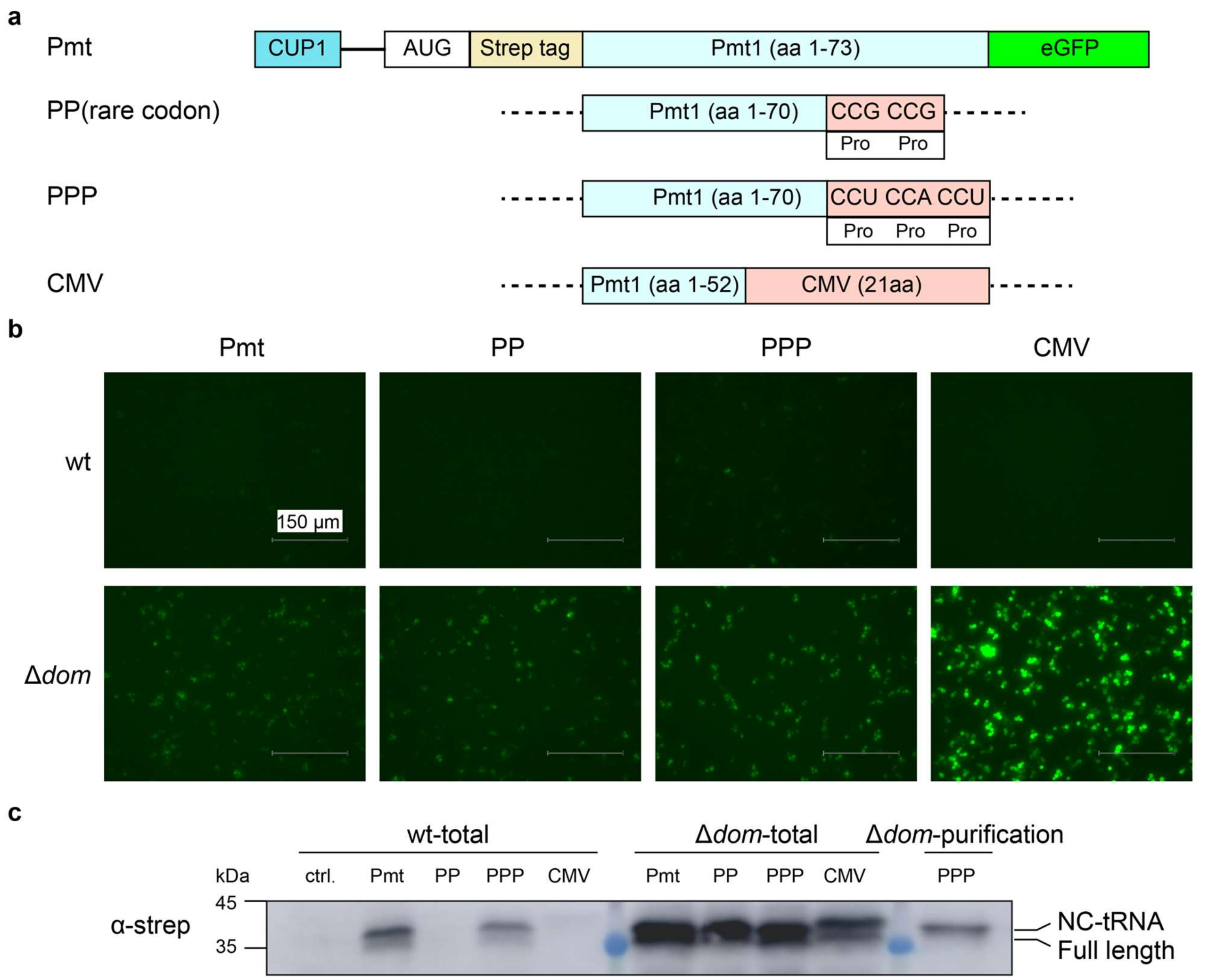

Supplementary Figure 5. Design and purification of RNC-RAC complexes.

a, Schematic diagram of the construct design for purifying RNCs. CUP1, Cu^{2+} induced promoter; Pmt, amino acids sequence of Pmt1 (aa 1-73); PP, a proline di-peptide encoded by two rare codons; PPP, a proline tri-peptide encoded by three canonical codons; CMV, cytomegalovirus arrest peptide.

b, Expression tests of different constructs. Representative live cell eGFP imaging of yeast cells after induced by 100 μM $CuSO_4$ for 0.5 h (cultured to OD ~1, induced and diluted in ten folds).

c, Western blotting examination of cell extracts in **b** (exposure time 0.1s). Freshly purified RNCs from the Δ*dom*-PPP strain were also examined. NC-tRNA, nascent chain-tRNA.

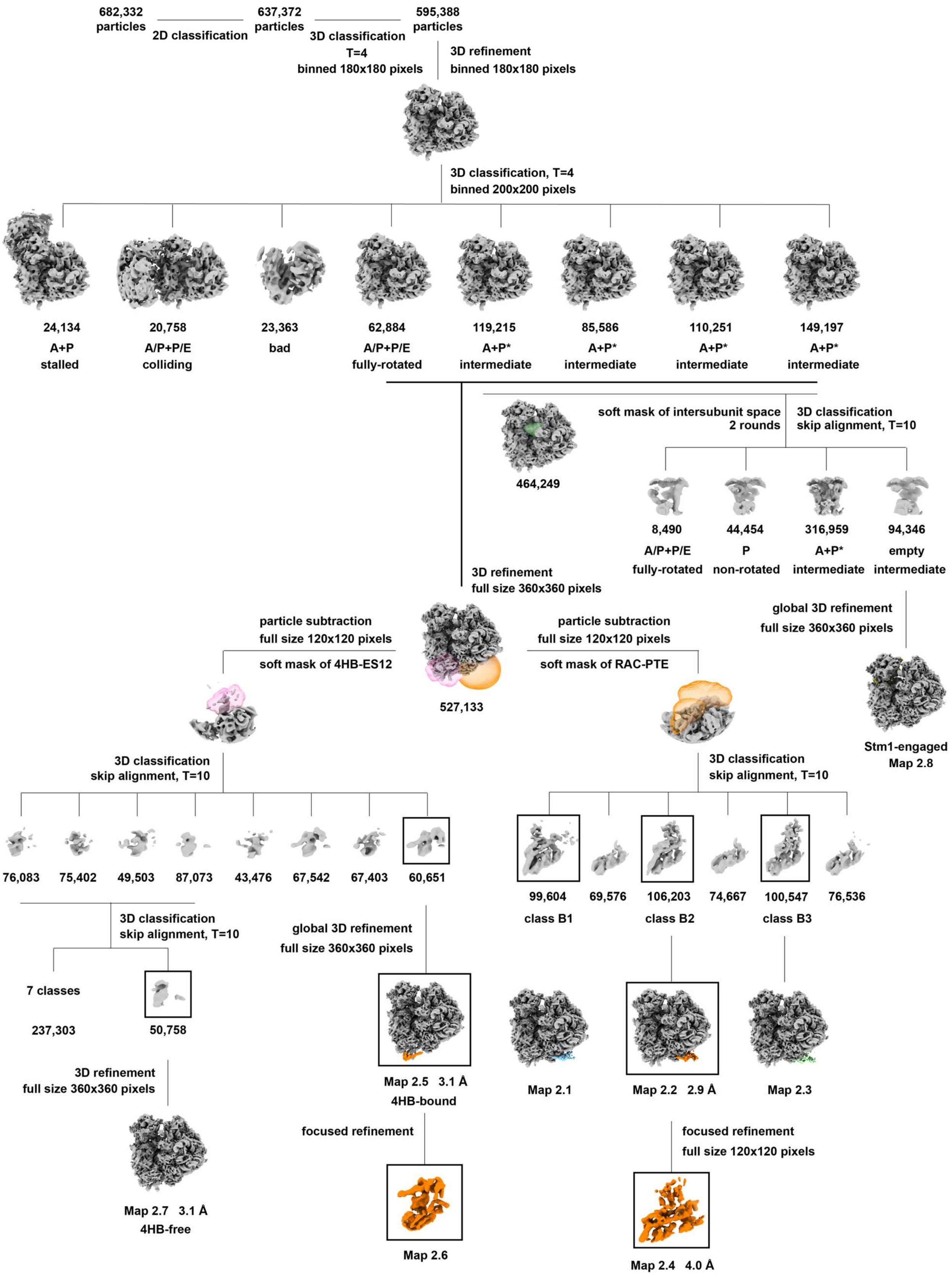

Supplementary Figure 6. Particle classification and refinement of the RNC-RAC-Ssb1 dataset.

Image processing was performed with RELION3.1. 2D and 3D classification were used to discard non-ribosomal and bad particles. See methods for details.

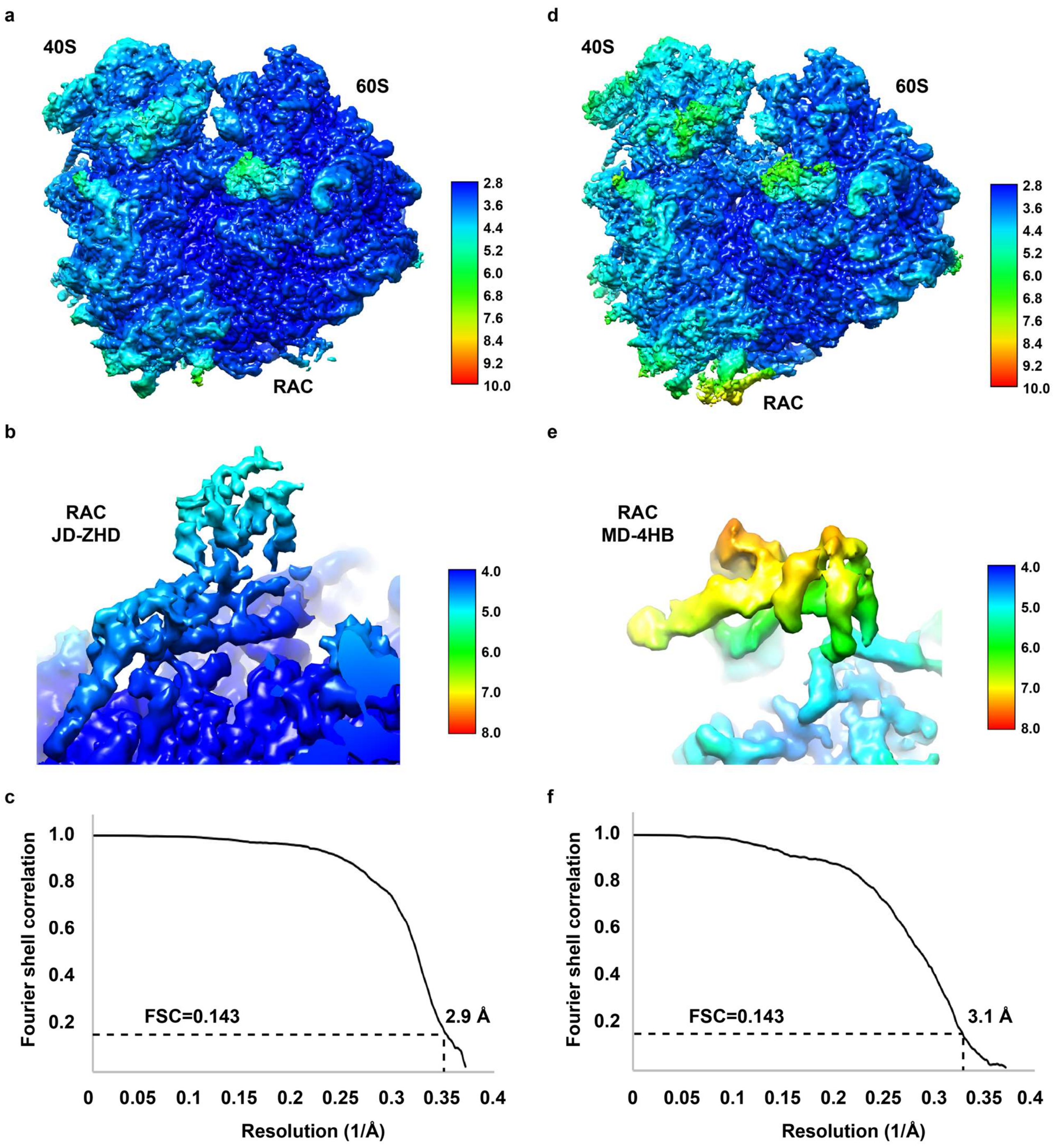

Supplementary Figure 7. Local resolution and FSC curves of the cryo-EM maps of the RNC-RAC-Ssb1 dataset.

a, Local resolution map of Map 2.2 obtained from the global refinement.

b, Local resolution map of the RAC region in Map 2.4 obtained from the focused refinement.

c, Fourier Shell Correlation (FSC) curves for Map 2.2 (2.9 Å) using the gold standard FSC 0.143 criteria.

d, Local resolution map of Map 2.5 obtained from the global refinement.

e, Local resolution map of the 4HB-ES12 region in Map 2.6 obtained from the focused refinement.

f, FSC curve for Map 2.5 (3.1 Å) using the gold standard FSC 0.143 criteria.

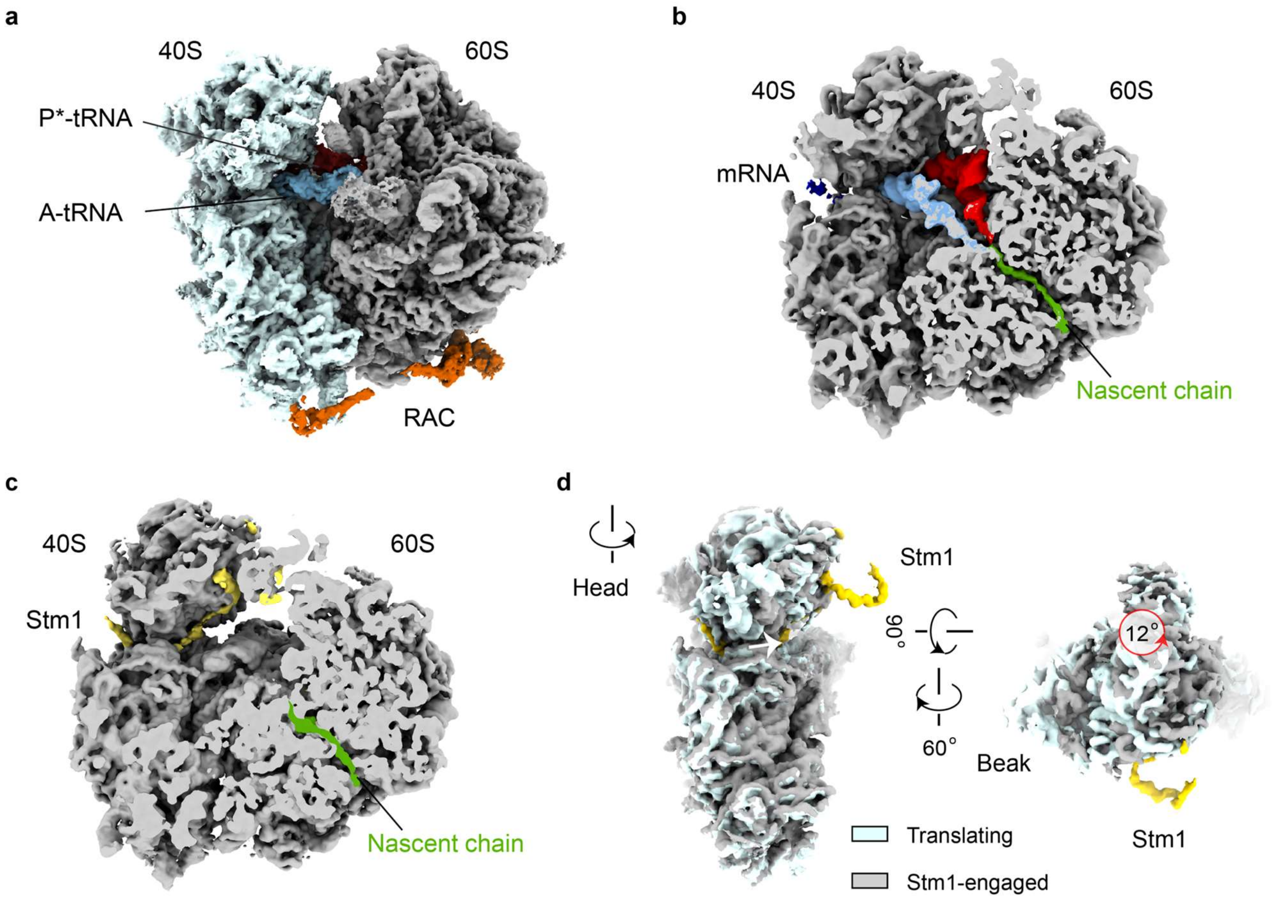

Supplementary Figure 8. Overview of the translating RNCs from the RNC-RAC-Ssb1 dataset.

a, The composite map of a representative translating ribosome from the RNC-RAC-Ssb1 dataset. The density map of the 40S subunit is from Map 2.5 and the 60S subunit plus tRNAs from Map 2.2 in Supplementary Fig. 6. The 40S and 60S subunits are colored light cyan and gray, respectively. A-site tRNA, P*-site tRNA and RAC are shown in light blue, red and orange respectively. P*-tRNA indicates a slight twist of the CCA arm towards the E site compared to the P/P-tRNA.

b, A cross-section view of a translating RNC structure (Map 2.2 in Supplementary Fig. 6) is also shown to highlight the Pmt1 nascent chain (colored green) in the peptide exit tunnel.

c, A cross-section view of Stm1-engaged RNC with no tRNA binding (Map 2.8 in Supplementary Fig. 6). Nascent chain and Stm1 are colored green and gold, respectively.

d, Comparison of the density maps of the translating RNC and Stm1-engaged RNC in **b** and **c**. The two states were aligned using the 60S subunit as the reference. The 40S subunit of the actively-translating RNC is colored light cyan and that of the Stm1-engaged RNC gray. A swiveling of the 40S head (~12°) is seen in the Stm1-engaged RNC.

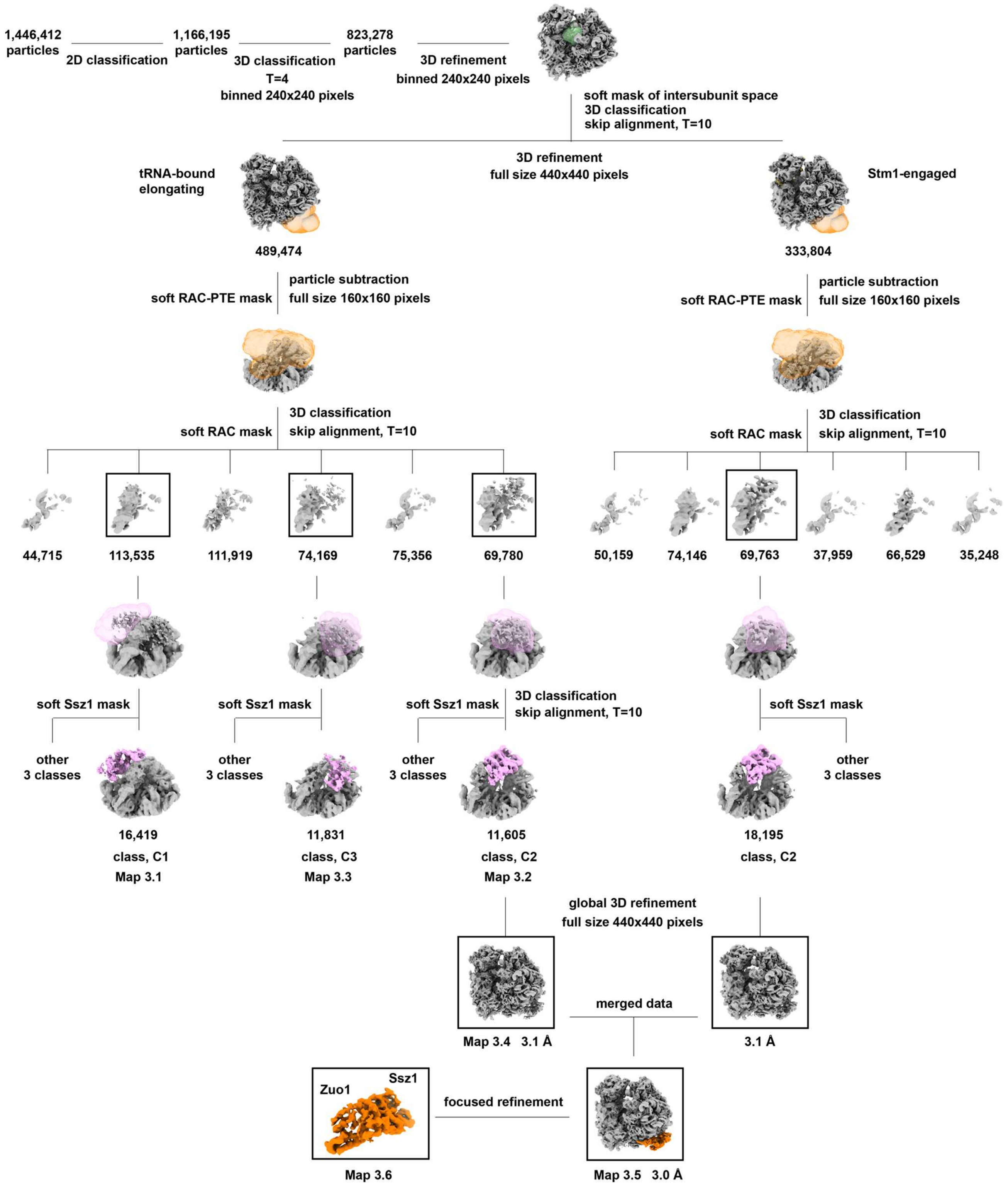

Supplementary Figure 9. Particle classification and refinement of the RNC-RAC dataset.

Image processing was performed with RELION3.1. 2D and 3D classification were used to discard non-ribosomal and bad particles. See methods for details.

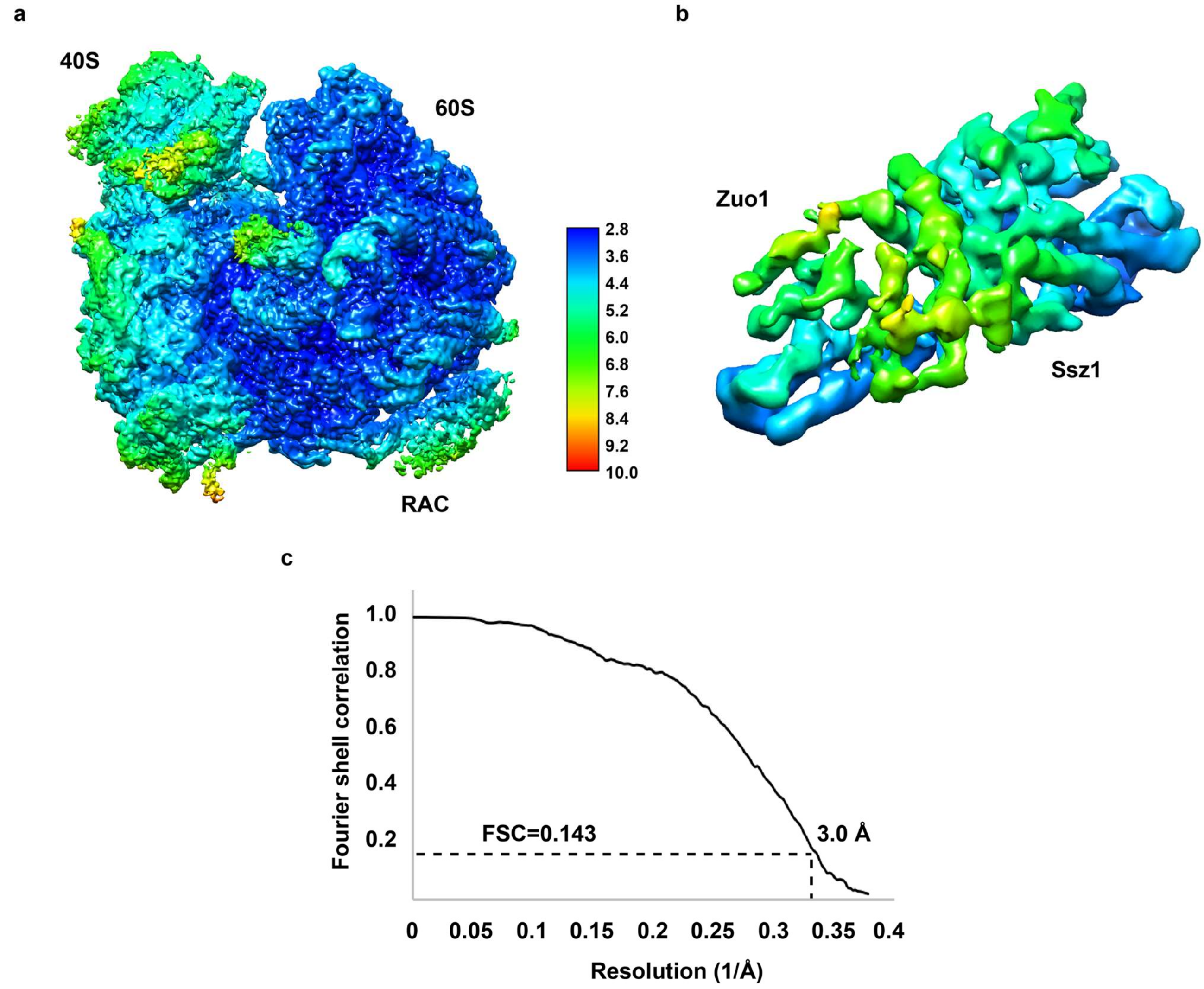

Supplementary Figure 10. Local resolution and FSC curves of the cryo-EM maps from the RNC-RAC dataset.

a, Local resolution map of State C2 of the RNC-RAC complex (Map 3.5) obtained from the global refinement.

b, Local resolution map of the RAC region (Map 3.6) obtained from the focused refinement.

c, Fourier Shell Correlation (FSC) curves for Map 3.5 (3.0 Å) using the gold standard FSC 0.143 criteria.

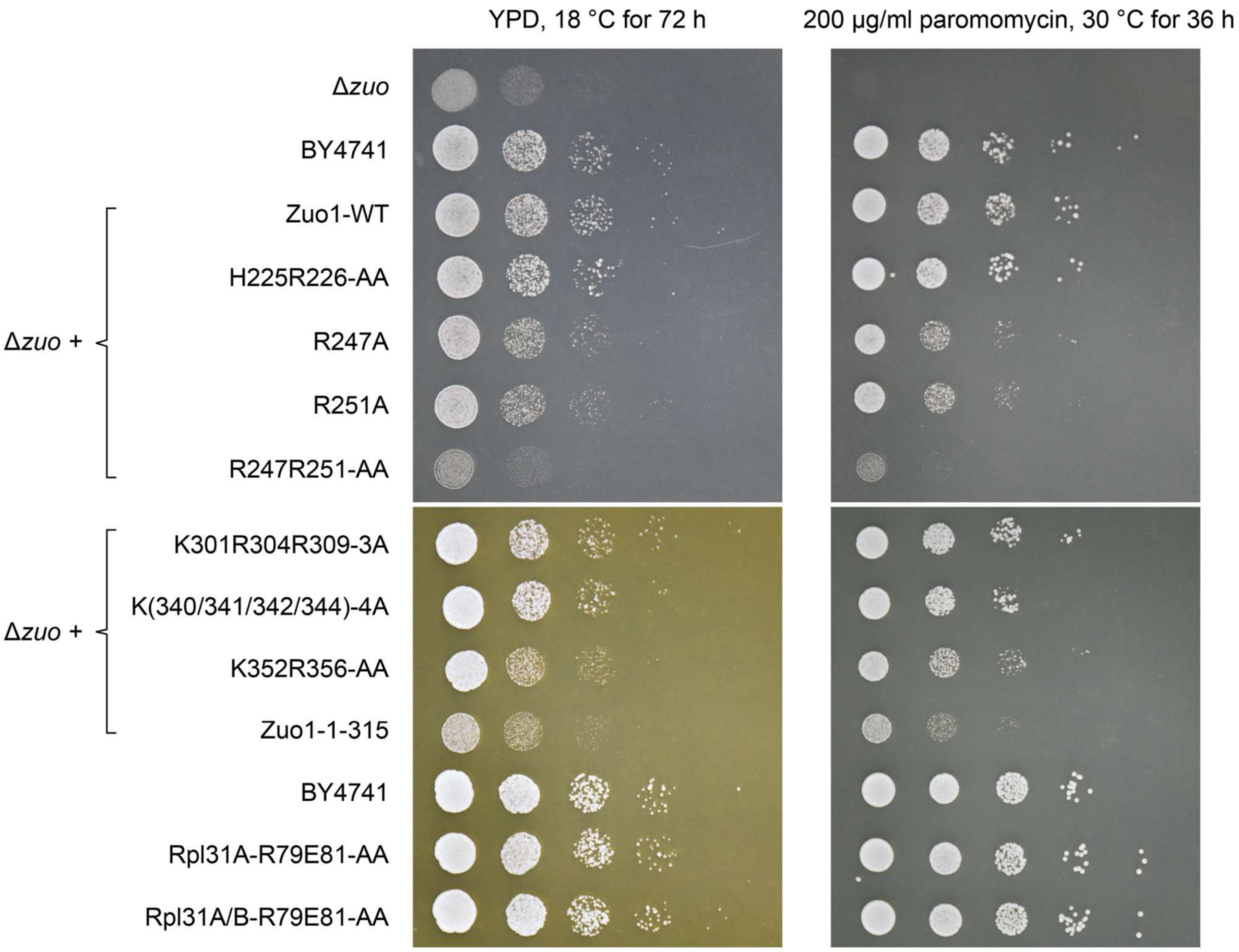

Supplementary Figure 11. Growth analysis of Zuo1 mutants bearing mutations in the ribosome-interacting regions.

Cells of different strains were cultured to an OD_{600} of ~0.8 and subjected to spot assay. Ten-fold serial dilutions of log-phase yeast cells were spotted on YPD plates and incubated at 18°C for 72 h, or on paromomycin-containing (200 μg/ml) YPD plates and incubated at 30°C for 36 h. The ZUO1 deletion strain (Δ*zuo*) was transformed with various single-copy pRS315 plasmids, encoding wild type Zuo1 (Zuo1-WT) or Zuo1 mutants (mutations and truncation).

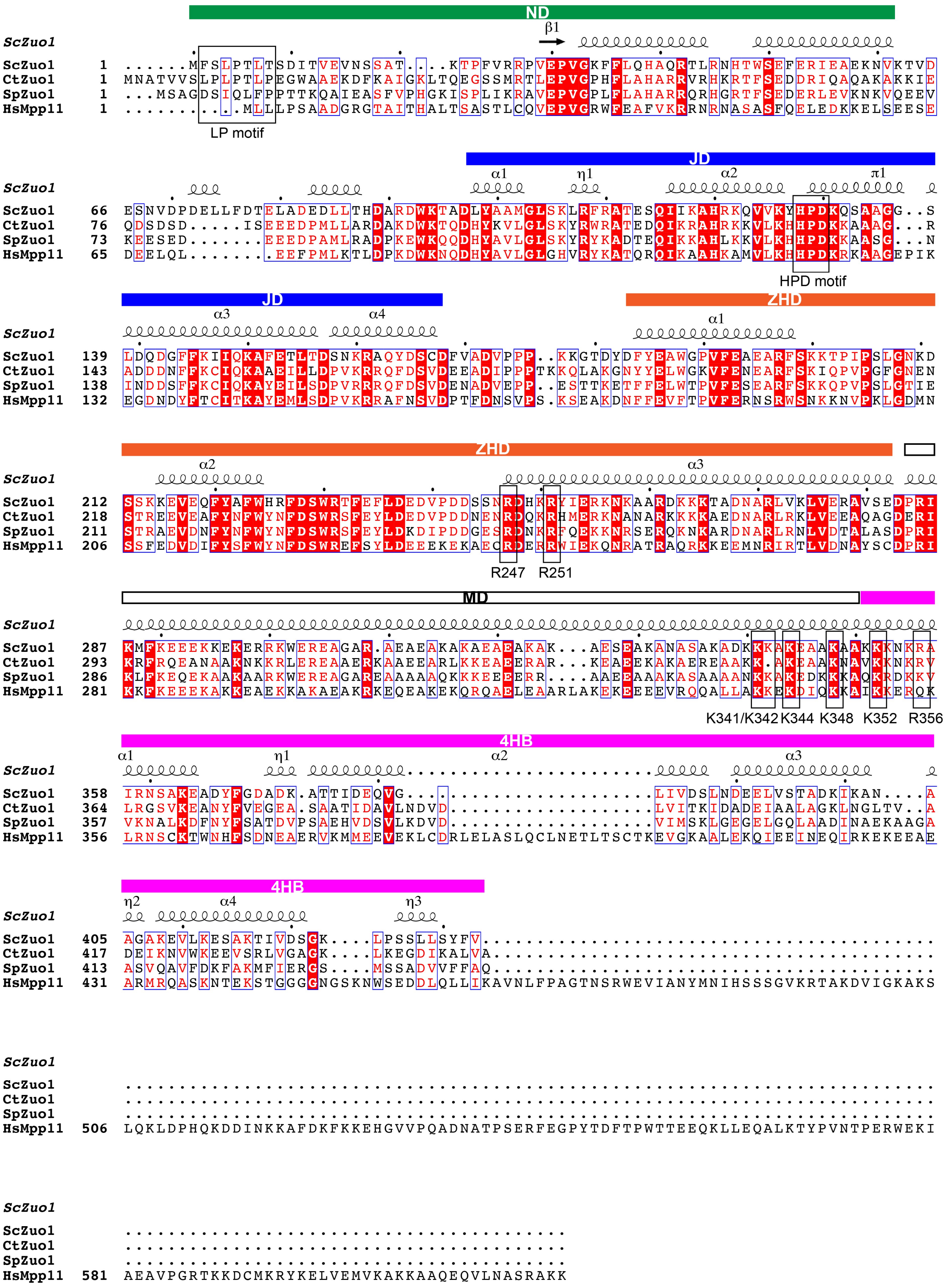

ND
β1
ScZuo1
ScZuo1 1 ......MFSLPTLTSDITVEVNSSAT.....KTPFVRRPVEPVGKFFLQHAQRTLRNHTWSEFERIEAEKNVKTVD
CtZuo1 1 MNATVVSLPLPTLPEGWAAEKDFKAIGKLTQEGSSMRTLEPVGPHFLAHARRVRHKRTFSEDDRIQAQAKAKKIE
SpZuo1 1 ...MSAGDSIQLFPPTTKQAIEASFVPHGKISPLIKRAVEPVGPLFLAHARRQRHGRTFSEDERLEVKNKVQEEV
HsMpp11 1 ..........MLLLPSAADGRGTAITHALTSASTLCQVEPVGRWFEAFVKRRNRNASASFQELEDKELSEESE
LP motif
JD
α1 η1 α2 π1
ScZuo1
ScZuo1 66 ESNVDPDELLFDTELADEDLLTHDARDWKTADLYAAMGLSKLRFRATESQIIKAHRKQVVKYHPDKQSAAG..S
CtZuo1 76 QDSDSD.....ISEEEDPMLLARDAKDWKTQDHYKVLGLSKYRWRATEDQIKRAHRKKVLKHHPDKKAAAG...R
SpZuo1 73 KEESED.......EEEDPAMLRADPKEWKQQDHYAVLGLSKYRYKADTEQIKKAHLKKVLKHHPDKKAASG...N
HsMpp11 65 DEELQL........EEFPMLKTLDPKDWKNQDHYAVLGLGHVRYKATQRQIKAAHKAMVLKHHPDKRKAAGEPIK
HPD motif
JD
ZHD
α3 α4 α1
ScZuo1
ScZuo1 139 LDQDGFFKIIQKAFETLTDSNKRAQYDSCDFVADVPPP..KKGTDYDFYEAWGPVFEAEARFSKKTPIPSLGNKD
CtZuo1 143 ADDDNFFKCIQKAAEILLDPVKRRQFDSVDEEADIPPPTKKQLAKGNYYELWGKVFENEARFSKIQPVPGFGNEN
SpZuo1 138 INDDSFFKCIQKAYEILSDPVRRRQFDSVDENADVEPP..ESTTKETFFELWTPVFESEARFSKKQPVPSLGTIE
HsMpp11 132 EGDNDYFTCITKAYEMLSDPVKRRAFNSVDPTFDNSVPS.KSEAKDNFFEVFTPVFERNSRWSNKKNVPKLGDMN
ZHD
α2 α3
ScZuo1
ScZuo1 212 SSKKEVEQFYAFWHRFDSWRTFEFLDEDVPDDSSNRDHKRYIERKNKAARDKKKTADNARLVKLVERAVSEDPRI
CtZuo1 218 STREEVEAFYNFWYNFDSWRSFEYLDEDVPDDNENRDQKRHMERKNANARKKKKAEDNARLRKLVEEAQAGDERI
SpZuo1 211 STRAEVDNFYNFWYNFDSWRSFEYLDKDIPDDGESRDNKRFQEKKNRSERQKNKARDNARLRNLVDTALASDPRI
HsMpp11 206 SSFEDVDIFYSFWYNFDSWREFSYLDEEEKEKAECRDERRWIEKQNRATRAQRKKEEMNRIRTLVDNAYSCDPRI
R247 R251
MD
ScZuo1
ScZuo1 287 KMFKEEEKKEKERRKWEREAGAR.AEAEAKAKAEAEAKAK...AESEAKANASAKADKKKAKEAAKAAKKNKRA
CtZuo1 293 KRFRQEANAAKNKKRLEREAAERKAAEEARLKKEAEERAR...KEAEEKAKAEREAAKK.AKEAAKNAVKKNKRV
SpZuo1 286 KLFKEQEKAAKAARKWEREAGAREAAAAAQKKKEEEERR....AAEEAAAKASAAAANKKAKEDKKKAQKRDKKV
HsMpp11 281 KKFKEEEKAKKEAEKKAKAEAKRKEQEAKEKQRQAELEAARLAKEKEEEEVRQQALLAKKEKDIQKKAIKKERQK
K341/K342 K344 K348 K352 R356
4HB
α1 η1 α2 α3
ScZuo1
ScZuo1 358 IRNSAKEADYFGDADK.ATTIDEQVG.......................LIVDSLNDEELVSTADKIKAN....A
CtZuo1 364 LRGSVKEANYFVEGEA.SAATIDAVLNDVD...................LVITKIDADEIAALAGKLNGLTV..A
SpZuo1 357 VKNALKDFNYFSATDVPSAEHVDSVLKDVD...................VIMSKLGEGELGQLAADINAEKAAGA
HsMpp11 356 LRNSCKTWNHFSDNEAERVKMMEEVEKLCDRLELASLQCLNETLTSCTKEVGKAALEKQIEEINEQIRKEKEEAE
4HB
η2 α4 η3
ScZuo1
ScZuo1 405 AGAKEVLKESAKTIVDSGK....LPSSLLSYFV..
CtZuo1 417 DEIKNVWKEEVSRLVGAGK....LKEGDIKALVA...
SpZuo1 413 ASVQAVFDKFAKMFIERGS....MSSADVVFFAQ...
HsMpp11 431 ARMRQASKNTEKSTGGGGNGSKNWSEDDLQLLIKAVNLFPAGTNSRWEVIANYMNIHSSSGVKRTAKDVIGKAKS
ScZuo1
ScZuo1 ....
CtZuo1 ....
SpZuo1 ....
HsMpp11 506 LQKLDPHQKDDINKKAFDKFKKEHGVVPQADNATPSERFEGPYTDFTPWTTEEQKLLEQALKTYPVNTPERWEKI
ScZuo1
ScZuo1 ....
CtZuo1 ....
SpZuo1 ....
HsMpp11 581 AEAVPGRTKKDCMKRYKELVEMVKAKKAAQEQVLNASRAKK

Supplementary Figure 12. Multiple-sequence alignment of Zuo1 and annotations to the structure of *Sc*Zuo1.

Highly conserved sites for inter- or intra-molecular interactions of Zuo1 are labelled: the Leu-Pro rich (LP) motif in the N-terminus; the HPD motif of J-Domain; R247/R251, two conserved arginine residues essential for the binding to H24 of the 25S rRNA; K341/ K342/ K344/ K348, lysine cluster in the MD important for the binding to ES12 of the 18S rRNA; K352/R356, interacting residues in the 4HB-α1 for ES12. Ct, *Chaetomium thermophilum*; Sc, *Saccharomyces cerevisiae*; Sp, *Schizosaccharomyces pombe*; Hs, *Homo sapiens*. ND, N-terminal domain; JD, J-Domain; ZHD, Zuo1 homology domain; MD, highly charged middle domain; 4HB, four-helix bundle domain. β, β-strand; α, α-helices; η, 3 10-helices; π, π-helices. The alignment is color coded by conservation: strictly conserved residues are in red boxes, similar residues are in red font, and globally similar residues are in blue frames. Sequence alignments were performed using CLUSTALW and visualized with ESPript 3.0 (Robert and Gouet, 2014).

Supplementary Table 1. Cryo-EM data collection, refinement and validation statistics

	**80S-RAC (Map 1.2; EMD-32975)**	**RNC-RAC-Ssb1 dataset (Map 2.5, 4HB; EMD-32978)**	**RNC-RAC-Ssb1 dataset (Map 2.2, JD-ZHD; EMD-32977)**	**RNC-RAC dataset (Map 3.4; EMD-32988)**	**RAC (Map 3.6, local refinement; EMD-32991, PDB 7X3K)**
**Data collection and processing**					
Magnification	130,000	105,000	105,000	81,000	81,000
Voltage (kV)	300	300	300	300	300
Electron exposure (e^{-}/Å^{2})	58	35	35	47	47
Defocus range (μm)	1.0-1.9	1.0-1.6	1.0-1.6	0.8-1.6	0.8-1.6
Pixel size (Å)	1.055	1.356	1.356	1.07	1.07
Symmetry imposed	C1	C1	C1	C1	C1
Initial particle images (no.)	145,398	595,388	595,388	823,278	823,278
Final particle images (no.)	23,667	65,816	115,246	11,605	29,800
Map resolution (Å)	3.4	3.1	2.9	3.3	6
FSC threshold	0.143	0.143	0.143	0.143	0.143
Map resolution range (Å)	3.1-10.0	2.9-8.0	2.8-6.0	3.0-8.0	4-7
**Validation**					
MolProbity score					1.44
Clashscore					4.94
Poor rotamers (%)					0.00
Ramachandran plot					
Favored (%)					96.97
Allowed (%)					3.03
Disallowed (%)					0.00
