# Supplementary material for: Structural remodeling of ribosome associated Hsp40-Hsp70 chaperones during co-translational folding
Source: Nat Commun. 2022 Jun 14;13:3410. doi: 10.1038/s41467-022-31127-4 (PMC9197937; doi:10.1038/s41467-022-31127-4)
Supplement: Supplementary file 3 — Description of Additional Supplementary Files [file 41467_2022_31127_MOESM3_ESM.pdf]

File name: Supplementary Data 1

Description: The raw data files of the mass spectrometry analyses of the 80S-RAC and RNC-RAC samples as indicated in Supplementary Fig. 1.

File name: Supplementary Movie 1

Description: Structure of RAC in the C2 state from the RNC-RAC dataset. The pseudoatomic model of RAC, superimposed with segmented map of RAC from the C2 state of the RNCRAC dataset. Individual components are separately colored. The two conformations of the H7-loop of the 25S rRNA are shown.
